# Mitochondrial DNA Copy Number and Risk of Oral Cancer: A Report from Northeast India

**DOI:** 10.1371/journal.pone.0057771

**Published:** 2013-03-04

**Authors:** Rosy Mondal, Sankar Kumar Ghosh, Javed Hussain Choudhury, Anil Seram, Kavita Sinha, Marine Hussain, Ruhina Shirin Laskar, Bijuli Rabha, Pradip Dey, Sabitri Ganguli, Monisha NathChoudhury, Fazlur Rahman Talukdar, Biswadeep Chaudhuri, Bishal Dhar

**Affiliations:** Department of Biotechnology, Assam University, Silchar, Assam, India; Health Canada, Canada

## Abstract

**Background:**

Oral squamous cell carcinoma (OSCC) is the sixth most common cancer globally. Tobacco consumption and HPV infection, both are the major risk factor for the development of oral cancer and causes mitochondrial dysfunction. Genetic polymorphisms in xenobiotic-metabolizing enzymes modify the effect of environmental exposures, thereby playing a significant role in gene–environment interactions and hence contributing to the individual susceptibility to cancer. Here, we have investigated the association of tobacco - betel quid chewing, HPV infection, *GSTM1-GSTT1* null genotypes, and tumour stages with mitochondrial DNA (mtDNA) content variation in oral cancer patients.

**Methodology/Principal Findings:**

The study comprised of 124 cases of OSCC and 140 control subjects to PCR based detection was done for high-risk HPV using a consensus primer and multiplex PCR was done for detection of *GSTM1-GSTT1* polymorphism. A comparative ΔCt method was used for determination of mtDNA content. The risk of OSCC increased with the ceased mtDNA copy number (*P_trend_* = 0.003). The association between mtDNA copy number and OSCC risk was evident among tobacco – betel quid chewers rather than tobacco – betel quid non chewers; the interaction between mtDNA copy number and tobacco – betel quid was significant (*P* = 0.0005). Significant difference was observed between *GSTM1* - *GSTT1* null genotypes (*P* = 0.04, *P = *0.001 respectively) and HPV infection (*P*<0.001) with mtDNA content variation in cases and controls. Positive correlation was found with decrease in mtDNA content with the increase in tumour stages (*P*<0.001). We are reporting for the first time the association of HPV infection and *GSTM1-GSTT1* null genotypes with mtDNA content in OSCC.

**Conclusion:**

Our results indicate that the mtDNA content in tumour tissues changes with tumour stage and tobacco-betel quid chewing habits while low levels of mtDNA content suggests invasive thereby serving as a biomarker in detection of OSCC.

## Introduction

OSCC, the most frequent tumour of oral cavity, [1] and the sixth most common cancer globally that accounts for approximately 5 per cent of all malignant tumours worldwide [2], [3]. The statistical analysis by the International Agency for Research on Cancer (IARC) indicated that the lip and oral cavity is the tenth most common tumour site in the human [4]. Smokeless tobacco products and betel quid with or without tobacco are the major risk factors for oral cavity cancer in Taiwan, India, and other neighboring countries [5]–[7]. In Northeast India, incidence of tobacco related oral cancers is about 33% [8]. Smoking, alcohol use, smokeless tobacco products, and HPV (Human papilloma virus) infections are the major risk factors for oral cavity cancer, with smoking and alcohol having synergistic effects [9], [10].

The development of carcinogenesis due to environment-gene interaction has been well illustrated by phase I and phase II enzymes that are involved in the metabolism of carcinogens. The phase I enzymes are CYPs (Cytochrome P450) that are involved in activating the environmental procarcinogens adding or exposing their functional groups whereas phase II enzyme like GST (Glutathione S-transferase) are involved in detoxication of the activated metabolites of the carcinogens [11]. Tobacco smoke is a complex mixture of carcinogenic compounds, and smokeless tobacco is rich in nitrosamines. Furthermore, the concomitant use of betel quid leads to 50-fold increase in reactive oxygen species generation (ROS) [12], [13]. A structural deletion in these genes represents a null genotype and has been associated with an increased risk to oral cancer [14].

Mitochondrial defects have long been suspected to play an important role in the development and progression of cancer [15], [16]. Mitochondrial respiratory activity is associated with the generation of ROS. The mitochondrial genome is susceptible to ROS and other types of genotoxic damage due to lack of protective histones and its limited mtDNA repair capabilities. The mtDNA copy number per cell is maintained within a constant range to meet the energy requirement of the cell to sustain normal physiological functions. It varies significantly among the population from 1000 to 10,000 per cell [17] and also significantly varies by cell type. It is likely that the variations in the copy number of mitochondria reflect the net results of gene–environmental interactions between unknown hereditary factors and the levels of oxidative stress (an imbalance between ROS production and the antioxidant capacity), caused by a variety of endogenous and exogenous factors, such as, hormones, age, dietary and environmental oxidants/antioxidants, and reaction to oxidative damage, all of which are thought to be risk factors for various types of cancer development [18]–[20].

MtDNA content has been implicated as a potential biomarker for several cancer types [21], [22]. Decreased mtDNA content had been reported for thyroid [21], renal [23], [24], gastric [25], breast [26], previously-treated head and neck [27], ovarian [28] and hepatic cancer [29]. In contrast, several studies have revealed an increased mtDNA content in prostate [30], untreated head and neck [31], endometrial [32], lung [33], colorectal [34], [35] and pancreatic cancer [36].

The aim of the present study was to investigate the association of tobacco - betel quid chewing, HPV infection, *GSTM1-GSTT1* null genotypes, with mtDNA content. We also evaluated the mtDNA content in the tumour and correlated with tumour stages. OSCC is a multifactorial and dynamic event in which numerous alterations contribute to disease development. Therefore, the risk of tobacco - betel quid chewing, *GSTM1-GSTT1* null genotypes, HPV infection and mtDNA content associated with OSCC was studied which may serve as a possible molecular biomarker for early detection of oral cancer, being the most prevalent cancer of Northeast region of India.

## Materials and Methods

### Subjects and Sample Collection

One hundred twenty four OSCC patient’s post-treated tumour tissue/FFPE/oral swab and 140 non-OSCC (without cancer, having the habit of chewing tobacco-betelquid and also no family history of cancer) age and gender matched controls swab from inner cavity, was collected during July 2010 to August 2012 from hospitals as well as from home with written Informed consent and approved by IRB. The availability of such controls alone in the hospital was impossible as most of the patients come with their relatives does not fit within the criteria assigned for being controls in this present study. Data regarding age, gender, occupation and nature of consuming tobacco-betel quid habit (smoking or smokeless) and alcohol intake from OSCC subjects was abstracted from hospital records and on personal interviews. All possible precautions were taken to avoid any cross-contamination while collecting as well as processing of the samples.

#### Ethics statement

The present study was approved [No: IRB/CCHRC/01/2010] by Institutional Review Board (IRB), Cachar Cancer Hospital and Research Centre (CCHRC) (http://cacharcancerhospital.org), Meherpur, Assam, India.

### DNA Isolation

DNA was isolated from preselected regions of tumour tissue, formalin fixed paraffin embedded tissue (FFPE) and oral swab. The tissues were digested in TES (50 mM Tris-HCl pH 7.4, 25 mM EDTA, 150 mM NaCl) buffer and incubated overnight at 55°C the tissue digests. The DNA was subsequently isolated by phenol/chloroform/isoamylalcohol method followed by ethanol precipitation and re-suspended in TE (10 mM Tris-HCl pH 8.0, 1 mM EDTA) buffer and stored at −20°C [37]. Bioline Isolate Genomic DNA minikit (Bioline, UK) was used for isolation of genomic DNA from FFPE tissues following manufacturer’s instructions.

### Multiplex PCR for *GSTM1* and *GSTT1*

Analysis for *GSTM1* -*GSTT1* gene polymorphism using *CYP1A1* gene as internal control was done by multiplex PCR. The forward (F) and reverse (R) primers used for the amplification *GSTT1* was F5’-TTCCTTACTGGTCCTCACATTCTC-3′ and R 5′-TCACGGGATCATGGCCAGCA-3′, *GSTM1* was F5’-GAACTCCCTGAAAAGCTAAAGC-3′ and R5’-GTTGGGCTCAAATATACGGTGG-3′, *CYP1A1* was F5’- ACTGCCACTTCAGCTGTCT and R5’-GCTGCATTTGGAAGTGCTC respectively [38], [39]. The PCR programme used for amplification was: initial denaturation step at 94°C for 2 mins; 30 cycles of denaturation at 94°C for 30s; annealing at 59°C for 45s and elongation at 72°C for 90s. The amplified product was observed in 1.5% agarose gel.

### HPV Detection and Genotyping

PCR amplification for HPV detection were carried out with consensus primers GP5+/GP6+ followed by subtype detection of HPV 16 and 18 [40], [41]. Reaction mixture without DNA template was used as a negative control and that with known DNA template was used as a positive control which yielded PCR products of expected results. PCR amplification was carried out with forty cycles. The PCR products were analyzed by electrophoresis on 2% agarose gel. A molecular weight marker of 50 bp was also run simultaneously to identify the molecular size of the PCR products.

### Quantitative Real Time PCR

The StepOne™ Real-Time PCR System (Applied Biosystems) was used to perform PCR amplification for mtDNA D-loop (C-tract) region. *GAPDH* was used as a ‘housekeeping gene’ to normalize all of the threshold cycle (Ct) values. The forward (F) and reverse (R) primers used for amplification of C-tract region was F5’ CAGGGTCATAAAGCCTAAATAG 3′ and R5’GAGGTAAGCTACATAAACTGTG3’ (109 bp) and GAPDH was F5’GAAATCCCATCACCATCTTCC 3′ and R5’ GAGCCCCAGCCTTCTCCATG 3′ (125 bp) respectively. For each 10 µl reaction, 1 µl of unknown DNA was amplified containing 0.5 µl of each primer (20 pmol/µl), 5 µl of 2X SYBR Green Mastermix (Applied Biosystems), and 3 µl nuclease free water. The real-time PCR conditions consisted of initial denaturation and Taq polymerase activation at 95°C for 10 minutes followed by 40 cycles of 95°C for 45 seconds, 54°C for 45 seconds, and 72°C for 1 minute and followed by a melting curve analysis. Each measurement was repeated in triplicate and a non-template control was included in each experiment.

To determine the quantities of mtDNA and nDNA present in samples, the average threshold cycle number (Ct) values of the nDNA and mtDNA were obtained from each case. The level of mtDNA was calculated using the delta Ct (ΔCt) of average Ct of mtDNA and nDNA (ΔCt = CtmtDNA-CtnDNA) in the same well as an exponent of 2 (2^−ΔCt^).

### Statistical Analysis

Medians and frequencies of selected characteristics were compared between cases and controls using the Mann-Whitney U test for continuous and the Pearson chi-square for all other categorical variables. MtDNA copy number was categorized into quartiles based on the distribution among controls. Odds ratios (OR) and 95% confidence intervals (CIs) were estimated using logistic regression models. A test for trend was calculated using the mtDNA copy number as a continuous variable. Non-parametric Mann-Whitney test was used to test if mtDNA content alteration in tumour is different in OSCC cases and controls with or without HPV infection, *GSTT1* and *GSTM1* null genotypes. *P*-values less than 0.05 are considered statistically significant.

## Results

**Figure 1:**
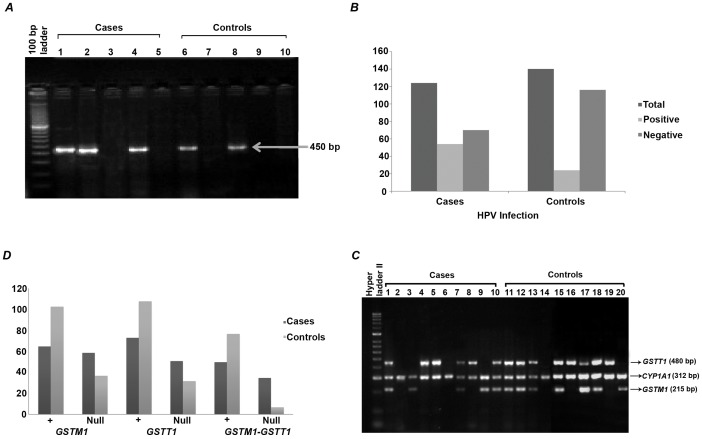
PCR based detection of HPV infection and *GST*s polymorphism in oral cancer. (A) Representative agarose gel stained with ethidium bromide for fragment size determination (at expected size of ∼450 base pairs) and polymerase chain reaction (PCR) amplification yield for common risk human papillomavirus (HPV) genomes from controls and oral cancer patients. (B) Bar graph showing the high incidence of HPV in OSCC patients than controls based on PCR detection of genomic DNA isolated from oral swab and tissues. (C) Multiplex PCR patterns for *GSTM1*, *GSTT1*, and *CYP1A* genes separated by agarose gel electrophoresis, corresponding to controls (lane 1–10) and oral cancer patients (lanes 11–20). The *CYP1A1* gene was used as an internal positive control. Lanes 1,4,5,7,10,11,12,13,15,11 and 18 represents the presence of both *GSTM1* and *GSTT1* genes, and lane 2, 6 and 14 represents null genotypes for both *GSTM1* and *GSTT1* genes. Lanes 3 and 20 represent presence of *GSTM1* gene and null genotypes for the *GSTT1*gene. Lanes 4, 58, 16 and 19 represent wild presence of *GSTT1* gene and null genotypes for the *GSTM1* gene. (D) Bar graph showing the distribution of *GSTM1* and *GSTT1* null genotypes among OSCC patients and controls based on multiplex PCR detection of genomic DNA isolated from oral swab and tissues.

The characteristics of the OSCC subjects and controls as described in Table 1. There were no statistically significant differences between the cases and controls subjects in terms of age (*P* = 0.82), gender (*P* = 0.2), intake of current fruit (*P = *0.94), salted dry fish (*P* = 0.77) and fermented fish (*P* = 0.84). However, significant differences were observed in daily tobacco-betel quid intake (*P* = 0.01) and vegetable (*P* = 0.04). The individual risk factors associated with oral cancer were examined. The increase risk to OSCC is 2.2- fold (95% CI, 1.31–3.68; *P* = 0.002) among the tobacco-betel quid chewers which is one of the major contributing factors for oral cancer and in Northeast India tobacco chewing is one of the customary practices. PCR of the oncogenic HPV genome was carried out using consensus primers GP5+/GP6+ and 450 bp band was observed (Figure 1A). The detection of common risk HPV in the individual was observed to be higher (Figure 1B) and was found 43.54% (54) in cases and 17.14% (24) in controls (Table 2). Upon genotyping HPV positive samples were found to be high risk subtype of HPV18. Infection with HPV has been implicated as one of the possible etiological factors for OSCC and in the present study, the risk of OSCC increased 3.72 -folds (95% CI, 2.11–6.56; *P*<0.0001) due to HPV infection.

**Table 1:** Selected characteristics of OSCC subjects and controls.

Characteristics	Subjects		*P* value
	Cases(n = 124) (%)	Control(n = 140) (%)	
**Age (years)**			
Median	58	56	0.82^a^
**Gender**			
Male	98(79)	101(72.1)	0.2^b^
Female	26(20.9)	39(27.8)	
**Current vegetable intake**			
<Once per week	23(18.5)	12(8.5)	0.04^b^
1–6 per week	61(49.1)	79(56.4)	
>1 per day	40(32.2)	53(37.8)	
**Current fruit intake**			
<Once per week	58(46.7)	63(45)	0.94^b^
1–6 per week	53(42.7)	61(43.5)	
>1 per day	13(10.4)	16(11.4)	
**Non-veg intake(fish)**			
**Salted Dry fish**			
<Once per week	12(9.7)	16(11.4)	0.77^b^
1–6 per week	63(50.8)	82(58.5)	
>1 per day	39(31.4)	42(30)	
**Fermented fish**			
<Once per week	18(14.5)	18(12.8)	0.84^b^
1–6 per week	71(57.2)	85(60.7)	
>1 per day	35(28.2)	37(26.4)	
**Daily tobacco- betel quid intake**			
No intake per day	35(28.2)	65(46.4)	0.01^b^
1–3 per day	50(40.3)	41(29.2)	
>3 per day	39(31.4)	34(24.2)	

a Mann - Whitney U was used to examine difference.

b Chi square was used to examine differences.

**Table 2:** Risk of tobacco and betelquid chewing, HPV and *GSTT1- GSTM1* null genotypes associated with OSCC.

	Cases(n = 124)	Controls(n = 140)	OR[95%CI]	*P* value
**Tobacco- betelquid**				
Chewers	89	75	2.20 [1.31–3.68]	0.002
Nonchewers	35	65	1(ref)	
**HPV**				
Presence	54	24	3.72 [2.11–6.56]	<0.0001
Absence	70	116	1(ref)	
***GSTM1***				
+	65	103	1(ref)	0.0003
Null	59	37	2.52 [1.50–4.22]	
***GSTT1***				
+	73	108	1(ref)	0.001
Null	51	32	2.35 [1.38–4.01]	
***GSTM1- GSTT1***				
+	50	77	1(ref)	<0.0001
Null	35	7	7.7 [3.17–18.67]	

Multiplex PCR was carried out among all the cases and the null genotype of *GSTT1* or *GSTM1* or both was detected by absence of the band when observed in 1.5% agarose gel (Figure 1C). We observed *GSTM1* null genotype in 47.58% (59) cases and 26.42% (37) controls, *GSTT1* null genotype in 41.12% (51) cases and 22.85% (32) controls, both *GSTT1* and *GSTM1* null genotypes were 28.22% (35) cases and 5% (7) controls respectively (Figure 1D). The *GSTM1* null genotype have increased oral cancer risk by 2.52 -fold (95% CI, 1.50–4.22; *P* = 0.0003) as null genotypes of this class gene have been linked with number of cancers, likely due to an increased susceptibility to environmental toxins and carcinogens, whereas the risk association of *GSTT1* null genotype with OSCC found to be statistically significant (OR, 2.35; 95% CI, 1.38–4.01; *P* = 0.001) (Table 2). Further the risk increases by 7.7-fold for OSCC with both *GSTM1-GSTT1* null genotypes (95% CI, 3.17–18.67; *P*<0.0001).

**Figure 2:**
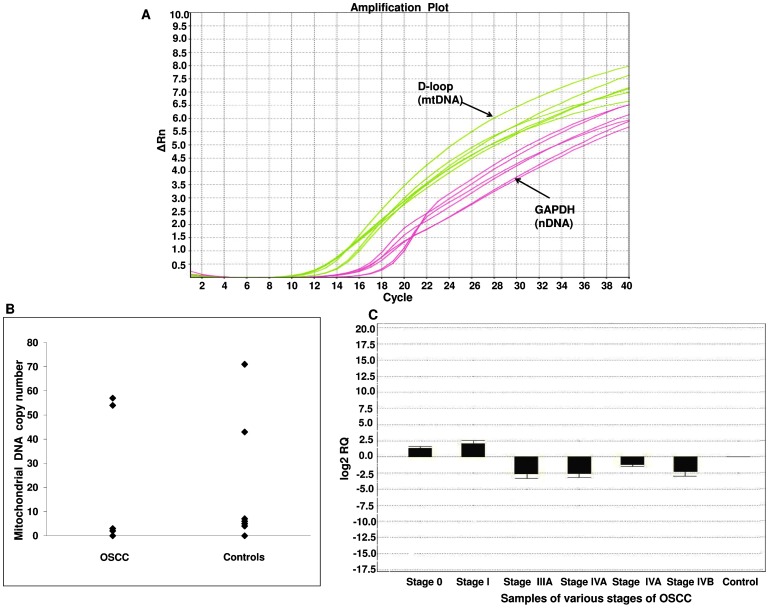
Real time PCR based mitochondrial DNA copy number determination in oral cancer. (A) Quantitative PCR of D-loop region and *GAPDH* gene (representative curve). D-loop region and *GAPDH* are ubiquitous genes found in the mitochondrial and nuclear genomes, respectively. Using quantitative PCR in samples from the patient and control, the relative mitochondrial content was calculated. (B) Distribution of mtDNA copy number in OSCC and controls. (C) Mitochondrial content decreases with increase in tumour stage: the various stages of OSCC samples from stage 0 to stage IVB with respect to log 2 RQ (log fold change).

Using quantitative PCR techniques, we determined the relative content of mtDNA with respect to the *GAPDH* gene in 124 OSCC patients with different tumour stage and in the normal oral mucosal cells in 140 individuals without disease (Figure 2A). Overall, the relative median of the mtDNA content is significantly lower in cases (0.22 relative copies) than the controls (0.89 relative copies) (*P*<0.009). The distribution of mtDNA content in cases and controls was shown in Figure 2B. OSCC cases in the lowest quartile of the mtDNA copy number experienced a significantly increased risk of 2.92 fold to oral cancer (95% CI, 1.32–6.43) compared with those in the highest quartile (Table 3). We observed that risk of OSCC increased with the ceased mtDNA copy number (*P_trend_* = 0.003).

**Table 3:** ORs and 95% CI for mtDNA copy number and risk of oral cancer.

mtDNA copy number quartile	Cases (n = 124)	Controls (n = 140)	OR (95%CI)
Quartile 1 (≤0.1)	58	43	2.92[1.32–6.43]
Quartile 2 (>0.1–1)	35	42	1.80[0.79–4.09]
Quartile 3 (>1–10)	19	29	1.41[0.57–3.47]
Quartile 4 (>10)	12	26	1 (ref)
*P*_trend_ 0.003			

The association between mtDNA copy number and OSCC risk was evident among tobacco – betel quid chewers rather than tobacco – betel quid non chewers (Table 4); the interaction between mtDNA copy number and tobacco – betel quid was significant (*P* = 0.0005). Similar results were observed when cases and controls were classified as tobacco- betel quid chewers and non chewers based on low (≤1) and high (>1) mtDNA copy number, the tobacco–betel quid chewers with the low mtDNA copy number have 3.54 fold increased risk of OSCC (95% CI, 1.59–7.87). Furthermore, a significant difference was found with mtDNA content in cases and controls with or without HPV infection (*P*<0.001). Similarly, we found a significant difference between *GSTM1* and *GSTT1* null genotypes with mtDNA content in cases and controls (*P* = 0.04 and *P = *0.001 respectively).

**Table 4:** mtDNA copy number and risk of oral cancer stratified by tobacco-betel quid chewing.

mtDNA content	Tobacco- betel quid Chewers		Tobacco- betel quid Non-chewers		*P*-interaction
	Cases/Controls	OR (95%CI)	Cases/Controls	OR (95%CI)	
**low≤1**	69/46	3.54 [1.59–7.87]	24/39	1.45 [0.60–3.46]	0.0005
**high>1**	20/29	1.63 [0.65–4.03]	11/26	1(ref)	

The mtDNA contents in tumour tissues was significantly higher in stage 0 and 1 tumours than in stage IV tumours, *P<*0.001. Out of 124 cases 18.5% (23) were stage 0, 25% (31) of tumours were stage I, 27.4% (34) stage III, 29% (36) were stage IV respectively. There were no samples available at tumour stage II. The mtDNA content correlated with tumour stage, where we observed that mtDNA content decreases with the increase in tumour stage (*P*<0.001) (Figure 2C).

## Discussion

The habit of chewing tobacco and betelquid is an endemic habit throughout the Indian subcontinent. The betel quid is commonly referred to as ‘paan’ in South Asian countries. The main constituents of a betel quid are Piper betel leaves and areca nut (the seed of the Areca catechu plant). It is made by wrapping chopped areca nut in a Piper betel leaf, and some lime (calcium hydroxide) and tobacco leaves or zarda (flavoured tobacco) may be included to improve the taste; combinations of ingredients are altered according to individual preferences. Tobacco consumption by smoking or chewing is thought to be the major etiological risk factors for the development of oral cancer caused by irritation from direct contact with the mucous membranes of mouth.

The elevated number of tobacco-related OSCC cases is a major concern Northeast region of India. All forms of tobacco produce free radicals that deplete antioxidants and cause oxidative damage to DNA, proteins and lipids [42], [43]. Antioxidant-rich foods such as green-leafy vegetables and fruits that may help reduce the oxidative stress caused by tobacco [44],[45] are usually lacking in the diet [46], [47], the reasons may be the poor socio-economic condition and also customary practice of oral consumption.

In the present study, we examined the high risk HPV infection, a known independent causative agent for oral cancer in OSCC patients and a significant difference with mtDNA content in cases and controls with or without HPV infection (*P*<0.001) is obtained. However, no reports of association of HPV infection with the mtDNA copy number are there, although the correlation of HPV infection with mitochondrial mutation was reported in a study of cervical cancer [48]. Bak protein is pro-apoptotic member which localizes in mitochondria, and functions to induce apoptosis. The elimination of Bak protein by HPV *E6* promotes survival of HPV infected cells by delaying apoptosis thereby facilitating tumour development with corresponding variation to mtDNA content, for which the exact mechanism is yet to be revealed. We are reporting for the first time the association of HPV infection with mtDNA content variation.

A significant difference between *GSTT1* and *GSTM1* null genotypes with mtDNA content in cases and controls (*P* = 0.04 and *P = *0.001) was observed. The presence of both *GSTM1* and *GSTT1* are essential for detoxication of carcinogenic compound. The most important risk factor for oral cancer is smoking, tobacco chewing and betel quid. The concomitant use of betel quid leads to a 50-fold increase in reactive oxygen species generated [12]. The increased risk factor of null *GSTs* with accumulation of mtDNA mutations enzyme as because possibly plays inside the mitochondrial matrix as mtDNA protection factor regarding damage caused by reactive oxygen species which in turn affect the mtDNA content and may lead to causation of OSCC as well [39]. The associations of GST null genotypes and mtDNA content is not yet been reported.

Low levels of mtDNA copy number in tobacco- betel quid chewers found in our study are associated to high risk of OSCC due to release of substantial amounts of ROS. [49] which in turn increase mtDNA mutation in human oral tissues. The accumulation of mtDNA deletions and subsequent cytoplasmic segregation of these mutations during cell division could be important contributors to the early phase of OSCC [50], [51]. The depletion in mtDNA may be result of the repression of mitochondrial biogenesis. The mtDNA copy number in cancer probably depends on several factors, including the site of mutation in the mitochondrial genome as demonstrated in D-loop region, a highly susceptible site for oxidative damage compared with the other regions of mtDNA [52]. The findings of the present study well demonstrate the risk of OSCC and mtDNA copy number to tobacco-betel quid chewers in this region. We did not evaluate the cancer tissue specimens for mtDNA determination before treatment due to its non availability from the biorepositary. Thus, we could not determine the mtDNA changes before chemotherapy. This might be a limitation in this type of study, although it would offer us additional information.

The inverse correlation of mtDNA content correlated with histopathological tumour stage and observed in our study were supported by similar finding in post-treatment salivary rinses in head and neck squamous cell carcinoma [27]. However, decrease of mtDNA copy number in tumour tissues have been reported in a variety of human cancers, including HCC [53], [54], breast [55], [56], gastric [57], osteosarcoma [58] and other cancers [59], [60]. The underlying mechanism behind the low level of mtDNA content with increased tumour size is not clear. Furthermore, it was reported that decreased mtDNA content may result in decreased oxidative phosphorylation capacity that in turn may favor faster growth or increased invasiveness [61]. In general, decreased mitochondrial activity seems to be an adaptation to hypoxic environment of solid tumours during their development since low oxygen initiates lower oxidative stress under hypoxic conditions and hypoxia inducible factor (HIF) inhibits mitochondrial biogenesis [62] or disrupts mitochondria by mitophagy [63].When tumour is growing in size, cells are becoming more hypoxic, mitochondrial biogenesis is decreased [63]. Alternatively, the decrease of mtDNA posttreatment may reflect an effect of radiation that influences mtDNA content or mitochondrial number in cells, which may be responsible for reducing mtDNA.

The burden of oral diseases like oral cancer, periodontal disease, and tooth loss can be decreased by addressing common risk factors, which include avoiding smoking and consumption of tobacco related products and also intake of alcohol. Furthermore, practicing good oral hygiene like proper brush and floss daily along with routine cleaning and examination by the dentist can reduce the risk of oral diseases. The intake of fruits and vegetables can also protect against oral cancer as they are rich in antioxidants. HPV is one of the risk factor for oral cancer and the most reliable way to prevent infection with either high-risk or low-risk HPV is by avoiding any skin-to-skin oral, anal or genital contact with another person. Those who are sexually active, long term, term, mutually monogamous relationship with an uninfected partner is the strategy most likely to prevent HPV infection.

### Conclusion

Our results indicate that the mtDNA content in tumour tissues changes with tumour stage and tobacco-betel quid chewing habits. Significant deviation from the medium range is associated with poor survival. High levels of mtDNA content may indicate that tumours may undergo rapid tumour growth while low levels of mtDNA content suggests invasive thereby serving as a biomarker in detection of OSCC.
